# 1138. Utility of Methicillin-resistant *Staphylococcus aureus* Nares Screening in Hospitalized Children With Acute Infectious Disease Syndromes

**DOI:** 10.1093/ofid/ofab466.1331

**Published:** 2021-12-04

**Authors:** Ashley Sands, Nicole Mulvey, Denise E Iacono, Stefan Hagmann

**Affiliations:** 1 Cohen Children’s Medial Center, New Hyde Park, New York; 2 Long Island Jewish Medical Center, New Hyde Park, New York; 3 Cohen Children’s Medical Center, New Hyde Park, New York; 4 Donald and Barbara Zucker School of Medicine at Hofstra/Northwell, New Hyde Park, NY

## Abstract

**Background:**

Empirical antibiotic regimens frequently include treatment for methicillin-resistant *Staphylococcus aureus* (MRSA). Studies in adults with pneumonia support the use of a negative MRSA nares screening (MNS) to help de-escalate antibiotic therapy. Comparable pediatric data in the literature is scarce. We aimed to evaluate the use of MNS for antibiotic de-escalation in hospitalized children (< 18 years) at a tertiary children’s hospital.

**Methods:**

A retrospective chart review was conducted of pediatric inpatients (January 01, 2015 to December 31, 2020) with a presumed infectious diagnosis who had a PCR-based MNS test and a clinical culture (i.e. site of infection or blood) performed as part of their diagnostic work up. Those who were screened >5 days since admission or > 48 hours since start of MRSA-active antimicrobials, and those who had antibiotic treatment withdrawn after 48 hours because of negative cultures were excluded.

**Results:**

A total of 101 children were included with a median age (range) of 2 years (0-17) and about half (n=57, 56.4%) were male. Top three diagnosis groups were skin and soft tissue infections (n=33, 32.7%), toxin-mediated syndromes (n=21, 20.8%), and osteoarticular infections (n=13, 12.9%). Pneumonia accounted for only six (5.9%) patients. The prevalence of nasal MRSA colonization was 6.9% (n=7). The sensitivity of the MNS test to predict a MRSA infection was 42.9% with a specificity of 95.7%. The positive predictive value (PPV) and negative predictive values (NPV) were 42.9% and 95.7%, respectively. In about half (55/95, 57.9%) of patients initiated on anti-MRSA therapy, these agents were discontinued during the admission. A quarter (n=14, 25.5%) were de-escalated based on the negative MNS test alone, and another third (n=21, 38.2%) after negative MNS test and negative culture results became available.

**Conclusion:**

Pediatric providers at this institution have started to use the MNS to help limit anti-MRSA therapy. We noted a high NPV which suggests that MNS may be useful for timely de-escalation of anti-MRSA therapy and thereby a useful antimicrobial stewardship tool for hospitalized children. Prospective studies to evaluate the utility of MNS for the various infectious syndromes are warranted.

**Disclosures:**

**All Authors**: No reported disclosures

